# Individual and joint association of bioavailable testosterone and aging with neutrophil-to-lymphocyte ratio in Chinese middle-aged and elderly men

**DOI:** 10.1007/s40520-019-01333-0

**Published:** 2019-09-12

**Authors:** Jian Zhou, Yaping Wei, Yuan Lan, Jingjing Zuo, Xiangqing Hou, Weikai Hou

**Affiliations:** 1 Department of Endocrinology, Qilu Hospital of Shandong University, Jinan, 250012 Shandong People’s Republic of China; 2 Institute of Endocrinology and Metabolism, Shandong University, Jinan, 250012 Shandong People’s Republic of China; 3 Key Laboratory of Endocrinology and Metabolism, Shandong Province in Medicine and Health, No. 107 West Culture Road, Jinan, 250012 Shandong People’s Republic of China; 4 Department of Geriatrics, The First Affiliated Hospital of USTC, Division of Life Sciences and Medicine, University of Science and Technology of China, Hefei, 230001 Anhui People’s Republic of China; 5 Department of Preventive Medicine, School of Public Health and Management, Wenzhou Medical University, Wenzhou, 325035 Zhejiang People’s Republic of China; 6 School of Ophthalmology and Optometry, Wenzhou Medical University, Wenzhou, 325000 Zhejiang People’s Republic of China

**Keywords:** Chronic systematic inflammation, Neutrophil-to-lymphocyte ratio, Bioavailable testosterone, Aging

## Abstract

**Background and objectives:**

Accumulating evidences suggest that chronic systemic inflammation (CSI) is independently associated with large number of major non-communicable chronic diseases (NCDs) ranging from metabolic disorders to cancers, and neutrophil-to-lymphocyte ratio (NLR) has been accepted as a novel, convenient marker for CSI response. Testosterone deficiency in men is linked to high risk of NCDs. This cross-sectional study aimed to investigate the individual and joint association of bioavailable testosterone (BIOT) and aging with NLR.

**Methods:**

A total of 132 male adults were enrolled during Jan. 2011 and Oct. 2017 in the first affiliated hospital of University of Science and Technology of China. Local weighted regression (LOESS) and multivariable generalized linear regression models were utilized to comprehensively examine the individual and joint association between BIOT and age with NLR.

**Results:**

Obvious linear relationships between NLR and BIOT or age were observed with the LOESS models. NLR was negatively correlated to BIOT after adjusting for some potential confounding factors (*P* = 0.034). As compared to the lowest quartile of BIOT, the adjusted decrease of NLR for the 2nd, 3rd and 4th quartiles were 0.40, 0.64 and 0.72, respectively. Meanwhile, NLR was observed to be independently correlated to elevated age (*P* = 0.043). Furthermore, as compared to the counterparts, men over 70 years combined with plasma BIOT less than 4.7 nmol/L had the highest NLR level, which suggested that low BIOT and aging jointly correlated to the level of NLR (*P* = 0.005).

**Conclusion:**

BIOT deficiency and aging were individually and jointly correlated to CSI. Men over 70 years combined with BIOT < 4.7 nmol/L were more like to have higher grade of CSI than others.

## Introduction

Chronic systemic inflammation (CSI) has gained major attention in the past several decades and been widely accepted as a major contributor to large number of diseases ranging from metabolic disorders to cancers [1, 2]. CSI is believed to be one of the most important causes and playing an important role in the onset and development of diabetes, atherosclerosis and glycolipid disorders [3–6]. It is also reported to be associated with aging, obesity, disorder of lipid metabolism, poor cognition in children and a lot of major chronic, progressive and preventable diseases including cardiovascular diseases (CVD), diabetes mellitus (DM), cancers and others [7–11]. Since these major non-communicable chronic diseases (NCDs) can be managed upon early diagnosis, it enhances the necessity for a comprehensive approach in identifying the onset of CSI and related functional impairments in time. Nowadays, the role of inflammatory markers in NCDs has been extensively studied and consistent relationships between various inflammatory markers and NCDs have been well established in the past several decades [12, 13]. Nowadays, neutrophil-to-lymphocyte ratio (NLR) has been considered as an logical, inexpensive, easy to obtain, widely available prognostic marker of CSI as compared to various traditionally used markers such as absolute white blood cell (WBC) count, C-reactive protein (CRP), tumor necrosis factor alpha (TNF-α), interleukin 6 (IL-6) and others [13, 14].

Emerging evidences suggest that testosterone, the predominant androgen in men, has been shown to substantially decline throughout the aging process and is linked to several disease states including cardiac failure, ischemic heart disease and others in men [15, 16]. Recent studies suggest that testosterone can significantly restrict the release and expression of cytokines as well as chemokines [17] and lower testosterone in men is linked to the pathogenesis of the risk to develop CVD [18, 19]. Meanwhile, reduced level of testosterone is obviously associated with insulin resistance, visceral obesity and metabolic syndrome (MS), and plays a fundamental role in the onset and development of some NCDs [20]. Bianchi and colleagues report that testosterone administration in hypogonadal men greatly improves the regulation and development of MS in men, reduces the mortality risk and to restore normal level of testosterone is the primary treatment in men, along with caloric restriction and physical exercise [20].

Previous studies also report that the process of aging continuously produce inflammation mediums and increase their levels, which eventually lead to CSI status and are called as inflammageing [21–23]. One of the most accepted theory for this inflammageing is oxidative stress, which is also thought to participate in the endocrine system aging and collaborative endocrine system aging, promote the development of CSI [21].

As both NLR and testosterone are linked to the risk of many NCDs, it is necessary to assess their associations. However, both theoretical and applied research on this topic are rare. In addition, the modification due to aging on the association between testosterone and CSI has not been assessed either. In the present study, we sought to comprehensively investigate whether the bioavailable testosterone (BIOT), the primary active ingredient of testosterone, was linked to NLR and its joint effect with aging on NLR in Chinese middle-aged and older men.

## Materials and methods

### Study population

The participants of this study were enrolled from the routine physical examination of middle-aged population during January 2011 and October 2017 in the Department of Geriatrics in the first affiliated hospital of University of Science and Technology of China (USTC). The exclusion criteria were as follows: various acute infections, acute complications of diabetes, various malignant tumors, severe liver or renal dysfunction, hematological diseases, gonadal disease, adrenal disease, pituitary disease, autoimmune diseases as well as people received any testosterone or related hormone immunization agents in the past 3 months. Participants with one of the above exclusion criteria were eliminated from the current study. Furthermore, subjects with the total number of white blood cell lower than 4 mL or over 10 mL were also removed from the study population. Finally, this study included 132 male Han subjects, aged 70.9 ± 15.2 years old. The study protocol was approved by the institutional review boards at the first affiliated hospital of USTC. Written informed consents were obtained from each participant before the enrollment.

### Blood sample collection and laboratory assessment

Following 12 h of fasting, 10 mL venous blood sample was collected by venipuncture from each participant at 7–9 am. Of them, 2 mL of whole blood sample was used for the assessment of the count of leukocytes, neutrophils, lymphocytes and platelets within 1 h, using standard reagents and automated blood cell analyzer (Sysmex Coulter XS1800i, Japan) by a well-trained technician of the central laboratory in the first affiliated hospital of USTC. The other 8 mL of blood sample were averagely assigned into two parts: 4 mL of them with tubes containing sodium fluoride anticoagulant for plasma, another 4 mL without sodium fluoride anticoagulant for serum. They were separated within 30 min and stored at − 20 °C in a freezer for the following measurement by the same technician. Fasting plasma glucose (FPG), serum creatinine (Cr), blood urine nitrogen (BUN), uric acid (UA), albumin (ALB) and serum lipid profiles including total cholesterol (TC), triglyceride (TG), high-density lipoprotein cholesterol (HDL-C), low-density lipoprotein cholesterol (LDL-C) and very low-density lipoprotein cholesterol (VLDL-C) were assessed by the same technician using standard reagents and automatic biochemistry analyzer (HITACHI 7170S Japan). Glycosylated hemoglobin (HbA1c) detection was performed with high-performance liquid chromatography (MQ-2000PT, China). Serum C-peptide was measured by chemiluminescence method. The determination of serum sex hormone binding protein (SHBG) was conducted by electrochemiluminescence (Roche Cobas, E601, Switzerland). The assessment of total testosterone (TT) was performed based on the IMMULITE 2000 platform chemiluminescence immunoassays (Siemens, Germany). The concentration of 25-hydroxyvitamin D was detected using liquid chromatography–tandem mass spectrometry (LC–MS/MS), which has been accepted as the golden standard of 25-hydroxyvitamin D determination [24]. Free testosterone (FT) and BIOT were calculated by the free and BIOT calculator which was developed at the Hormonology Department, University Hospital of Ghent, Belgium (http://www.issam.ch/freetesto.htm). The concentration of 2-h postprandial plasma glucose (H2PG), serum insulin and C-peptide were determined after eating the standard 100 g steamed bread meal. Insulin resistance index was calculated by the formula of homeostatic model assessment (HOMA): HOMA-IR = fasting insulin (mIU/L) × fasting plasma glucose (mmol/L)/22.5 (https://en.wikipedia.org/wiki/Homeostatic model assessment). NLR was calculated as the ratio of the absolute neutrophil count by lymphocyte count.

### Covariates

Demographic characteristic information for each participant on gender, birth date, occupation, education, cigarette smoking, alcohol consumption, history of diseases and others were collected with face-to-face interview by trained investigator using a standardized questionnaire designed for this study. To avoid the impact due to potential information bias on the findings, all investigators received systematic training before the study and were asked to strictly follow the standard operation procedures (SOP) of this study. Effective measures were also taken to perform quality assurance and quality control during the current study.

### Statistical analysis

As no widely accepted medical reference ranges of BIOT as well as age could be found, multivariable local weighted regression (LOESS) model were utilized to obtain the fitted relation curves between NLR and BIOT or age to look for potential optimal cut-off values. The individual and joint associations of BIOT and age with NLR were separately performed using multivariable generalized linear regression models (GLMs). All the associations of BIOT and age with NLR were assessed in the following two ways: with categorical variable (quartile) for BIOT with the 1st quartile of BIOT concentration as the reference or three-level categorical variable for age with less than 60 years old as the reference group, and as a continuous variable scaled to standard deviation (SD). To evaluate the above-mentioned independent associations, we adjusted for associated potential confounding factors such as HbA1c, TG, LDL-C which were screened using stepwise regression models. Furthermore, the joint association of BIOT and age with NLR was also performed in a manner as BIOT classified with high BIOT determined as greater than the median and elder participants defined as higher than 70 years old. In addition, the interaction of BIOT and age on NLR was also investigated with a GLMs model. All tests were 2-sides and *P* ≤ 0.05 was set as the significant level. All the data management, data analysis and figures drawing were finished with Rstudio Version 1.1.423 (© 2009-2018 RStudio, Inc.).

## Results

### Characteristics of study population

A total of 132 male subjects aged 34–95 [median: 74.0, the 1st quartile (Q_1_): 60.0, the 3rd quartile (Q_3_): 83.0] years old were enrolled in the current study. They were averagely classified into two groups based on the serum BIOT level over 4.7 nmol/L (the median of BIOT) or not: among them, 65 and 67 participants with the BIOT concentration less than and equal to or greater than the median, respectively. Table 1 presents the demographic and clinical characteristics of these participants. As compared to their counterparts, male people with lower BIOT (< 4.7 nmol/L) tended to be older, higher level of HbA1c, H2PG, SHBP and the urinary albumin to creatinine ratio (UA/Cr). They were also more likely to have lower concentration of TG, lymphocyte number, ALB as well as UA, which indicated that age, HbA1c, H2PG, SHBP, UA/Cr, TG, lymphocyte, ALB and UA might be significantly associated with NLR. While, differences of other variables between the two groups did not reach the significant level (Table 1).

**Table 1:** Characteristics of study population

Variables	Overall (*N* = 132)	BIOT < 4.7 nmol/L (*N* = 65)	BIOT ≥ 4.7 nmol/L (*N* = 67)	*P* value
Age (years)	74.0 (60.0, 83.0)	82.0 (75.0, 86.0)	61.0 (52.0, 73.0)	< 0.001
HbA1c (%)	5.9 (5.4, 6.6)	6.1 (5.6, 7.2)	5.6 (5.3, 6.1)	< 0.001
Fasting plasma glucose (mmol/L)	5.3 (4.8, 6.3)	5.5 (4.9, 6.8)	5.3 (4.8, 5.9)	0.086
2-h postprandial glucose (mmol/L)	7.4 (6.0, 11.2)	9.7 (6.8, 12.1)	6.6 (5.5, 8.5)	< 0.001
Fasting plasma insulin (pmol/L)	67.1 (49.7, 102.0)	61.9 (47.1, 103.1)	73.0 (50.8, 102.0)	0.383
2-h postprandial insulin (pmol/L)	361.4 (235.3, 555.2)	362.3 (276.1, 675.7)	354.8 (197.8, 500.0)	0.324
Fasting plasma C-peptide (nmol/L)	0.5 (0.4, 0.7)	0.5 (0.4, 0.7)	0.6 (0.4, 0.7)	0.090
2-h postprandial C-peptide (nmol/L)	1.9 (1.4, 2.5)	1.9 (1.4, 2.5)	2.0 (1.4, 2.6)	0.532
HOMA-IR	2.4 (1.8, 3.7)	2.5 (1.7, 4.0)	2.4 (1.8, 3.6)	0.779
Total cholesterol (mmol/L)	4.5 ± 1.0	4.5 ± 1.1	4.6 ± 1.0	0.564
Triglyceride (mmol/L)	1.5 (1.0, 2.0)	1.2 (0.9, 1.9)	1.6 (1.0, 2.2)	0.012
High-density lipoprotein (mmol/L)	1.1 (0.9, 1.3)	1.1 ± 0.2	1.1 ± 0.2	0.636
Low-density lipoprotein (mmol/L)	2.5 (2.0, 3.0)	2.6 ± 0.9	2.6 ± 0.8	0.869
Very low-density lipoprotein (mmol/L)	0.7 (0.5, 1.0)	0.7 (0.5, 1.0)	0.7 (0.5, 1.0)	0.614
Lymphocyte (× 10^3^/mm^3^)	1.7 (1.4, 2.2)	1.6 (1.4, 1.9)	1.8 (1.5, 2.4)	0.004
Neutrophils (× 10^3^/mm^3^)	3.7 (2.8, 4.5)	3.8 (2.7, 4.5)	3.5 (3.0, 4.4)	0.902
Total white blood cell (× 10^3^/mm^3^)	5.9 (5.0, 7.3)	6.1 (5.0, 7.2)	5.9 (5.2, 7.5)	0.617
Sex hormone binding protein (nmol/L)	40.6 (27.5, 56.2)	49.9 ± 21.5	36.3 ± 17.3	< 0.001
Albumin (mg/dL)	40.9 ± 4.3	38.5 (36.3, 42.3)	43.4 (40.4, 45.0)	< 0.001
Creatinine (μmol/L)	81.0 (69.0, 92.6)	81.0 (70.0, 94.5)	80.0 (69.0, 91.0)	0.436
Blood urea nitrogen (mmol/L)	5.8 (4.9, 7.2)	6.1 (5.3, 7.5)	5.4 (4.8, 6.5)	0.032
Urinary acid (μmol/L)	369.4 ± 95.0	345.5 ± 101.6	392.9 ± 82.1	0.004
Urinary albumin/creatinine (mg/mmol)	13.6 (7.6, 24.1)	18.8 (11.0, 55.9)	10.4 (6.9, 19.6)	< 0.001
25(OH) vitamin D (nmol/L)	15.6 (11.5, 22.1)	15.5 (12.3, 21.1)	15.7 (10.9, 22.4)	0.961
Total testosterone (nmol/L)	11.1 (8.6, 14.8)	9.3 (6.9, 11.5)	13.8 (11.0, 16.5)	< 0.001
Estradiol (pmol/L)	132.0 (96.2, 168.0)	128.0 (94.0, 164.0)	135.0 (106.0, 178.0)	0.209
Follicle stimulating hormone (IU/L)	11.1 (6.3, 19.6)	15.7 (9.7, 29.2)	7.6 (5.1, 11.3)	< 0.001
Luteinizing hormone (IU/L)	6.7 (4.2, 9.8)	8.3 (5.3, 13.7)	5.0 (3.6, 7.3)	< 0.001

Continuous data were presented as mean ± standard deviation (SD) if they met normal or similar normal distribution and two independent samples *t* tests were performed the differences between the two groups, otherwise they were described as median [1st quartile (Q_1_), 3rd quartile (Q_3_)] and Mann–Whitney *U* tests were applied to compare the differences between the two groups

*BIOT* bioavailable testosterone, *HbA1c* glycated hemoglobin, *HOMA*-*IR* homeostasis model assessment-insulin resistance; *25(OH) vitamin D* 25-hydroxy vitamin D

### Individual association between bioactive testosterone and NLR

**Fig. 1:**
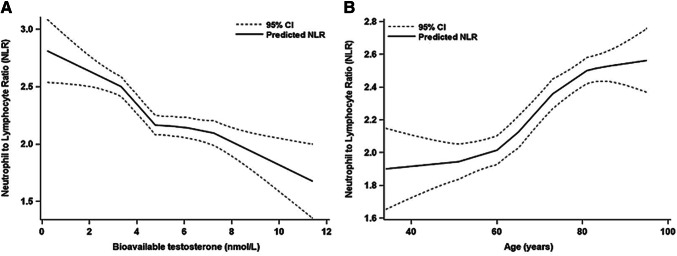
Relationship between the level of neutrophil to lymphocyte ratio (NLR) and bioavailable testosterone (BIOT) (**a**) as well as age (**b**) based on multivariable locally weighted regression (LOESS) model

Based on a multivariable LOESS model, an obvious dose–response relationship between BIOT concentration and NLR level was observed with a cut-off value of 4.7 nmol/L (Fig. 1). As compared to the lowest quartile, the adjusted average decrease of NLR were 0.33 ± 0.24 (*P* = 0.172), 0.53 ± 0.24 (*P* = 0.025) and 0.55 ± 0.25 (*P* = 0.030) for participants in the 2nd, 3rd, and 4th quartile of BIOT, respectively. NLR level was significantly and negatively correlated with serum BIOT concentration (*P* = 0.027) after adjusting for some potential confounding factors such as HbA1c, TG, LDL. Meanwhile, when the BIOT concentration was divided into two categories by the cut-off value (BIOT = 4.7 nmol/L) estimated with the LOESS model, the NLR levels for participants with lower (BIOT ≤ 4.7 nmol/L) and higher BIOT concentration (BIOT > 4.7 nmol/L) were 2.48 ± 1.18 and 2.09 ± 0.85, respectively. As compared to the counterparts, the adjusted NLR for participants with higher BIOT significantly decreased with 0.44 (*P* = 0.046). In addition, when the relationship between NLR and BIOT was examined as a continuous variable, the level of NLR decreased significantly by 0.23 with each standard deviation increase of BIOT after adjusting for the above-mentioned potential confounding factors (*P* = 0.034), which revealed significant negative relationship between NLR and BIOT concentration (Table 2).

**Table 2:** Relationship between neutrophils-to-lymphocyte ratio (NLR) and bioavailable testosterone based on multiple generalized linear regression models (GLMs)

Bioavailable testosterone (nmol/L)	*N*	Mean ± SD	Crude			Adjusted		
			*β*	SE	*P*	*β*	SE	*P*
Per SD = 2.05			− 0.20	0.09	0.022	− 0.23	0.11	0.034
Quartile								
Q_1_ (0.22–3.32)	32	2.67 ± 1.22	0.00	0.00	Ref.	0.00	0.00	Ref.
Q_2_ (3.33–4.76)	33	2.30 ± 1.12	− 0.37	0.25	0.135	− 0.40	0.29	0.169
Q_3_ (4.77–6.18)	34	2.05 ± 0.88	− 0.63	0.25	0.012	− 0.64	0.30	0.033
Q_4_ (6.19–11.40)	33	2.13 ± 0.84	− 0.54	0.25	0.030	− 0.72	0.31	0.021
Trend test					0.021			0.027
More than median (= 4.7)								
No	65	2.48 ± 1.18	0.00	0.00	Ref.	0.00	0.00	Ref.
Yes	67	2.09 ± 0.85	− 0.40	0.18	0.026	− 0.44	0.22	0.046

Adjusted for glycated hemoglobin, triglyceride, low-density lipoprotein; Q_1_, Q_2_, Q_3_ and Q_4_ indicate the 1st, 2nd, 3rd and 4th quartile, respectively

*SD* standard deviation

### Individual association between age and NLR

Figure 1b also shows an obvious positive relationship between NLR and age estimated with a multivariable LOESS model. Compared to subjects younger than 60 years old, the NLR level significantly increased by 0.55 in participants over 70 years old (*P* = 0.029) An obvious linear trend existed between NLR and age (*P* = 0.027). Meanwhile, the subjects were also classified into two groups with the elder group defined as older than 70 and the counterparts as less than 70 years old. The NLR level of the elder group significantly increased by 0.60 (*P* = 0.005), compared to the counterparts, after adjusting for HbA1c, TG, LDL. Furthermore, when the relationship between NLR and age was assessed as a continuous variable, the adjusted level of NLR significantly increased by 0.23 with each standard deviation increase of age after adjusting for the above-mentioned potential confounding factors (*P* = 0.043), which revealed a significant positive relationship between NLR and age (Table 3).

**Table 3:** Relationship between neutrophils-to-lymphocyte ratio (NLR) and age based on multiple generalized linear regression models (GLMs)

Age (years)	*N*	Mean ± SD	Crude			Adjusted		
			*β*	SE	*P*	*β*	SE	*P*
Strata I^a^								
34–59	32	1.99 ± 0.63	0.00	0.00	Ref.	0.00	0.00	Ref.
60–69	25	1.98 ± 0.98	− 0.01	0.27	0.980	− 0.09	0.28	0.747
70–95	75	2.51 ± 1.15	0.52	0.21	0.015	0.55	0.25	0.029
Trend test					0.009			0.027
Per SD = 15.14			0.24	0.08	0.005	0.23	0.11	0.043
More than 70^b^								
No	57	1.99 ± 0.80	0.00	0.00	Ref.	0.00	0.00	Ref.
Yes	75	2.51 ± 1.15	0.52	0.18	0.003	0.60	0.21	0.005

Adjusted for glycated hemoglobin, triglyceride, low-density lipoprotein

*SD* standard deviation

^a^The age was classified into three strata (34–59, 60–69 and 70–95) according to the widely accepted criteria for aging definition rather than quartiles

^b^As more and more elder people would be found in a population nowadays and 56.82% of participants were over 70 years, we select 70 as the cut-off value in estimating the relationship between NLR and age

### Joint association between serum BIOT concentration and age with NLR

Because the above results showed that serum BIOT and age were both independently correlated with the NLR level, it is necessary to further explore the joint association between serum BIOT concentration and age on the NLR based on multivariable GLMs. Mean ± SD of NLR were 1.99 ± 0.71 for subjects less than 70 years old and with BIOT ≥ 4.7 nmol/L (Category A),1.97 ± 1.16 for participants less than 70 years old and with BIOT < 4.7 nmol/L (Category B), 2.31 ± 1.11 for persons equal to and over 70 years old and with BIOT ≥ 4.7 nmol/L (Category C) and 2.58 ± 1.17 for population equal to or older than 70 years old and with BIOT < 4.7 nmol/L (Category D), respectively. The level of NLR was significantly increased with those older than 70 years old and the serum BIOT concentration < 4.7 nmol/L (Table 4) The highest increase of NLR was observed in people of category D as compared to subjects in category A, with the average raise of NLR by 0.68 after adjusting for the potential confounding factors such as HbA1c, TG, LDL-C. Age and serum BIOT significantly and jointly correlated with the level of NLR (*P* = 0.005) after adjusting for the impacts induced by the above-mentioned potential confounding factors. However, no significant interaction between age and serum BIOT concentration on NLR was observed in this study (*P* = 0.184) (Table 4).

**Table 4:** Joint association of age and bioavailable testosterone with neutrophil-to-lymphocyte ratio (NLR) based on multiple generalized linear regression model (GLMs)

Age (< 70 years)	Bioavailable testosterone (< 4.7 nmol/L)	*N*	Mean ± SD	Crude			Adjusted^a^		
				*β*	SE	*P*	*β*	SE	*P*
Yes	No	47	1.99 ± 0.71	0.00	0.00	Ref.	0.00	0.00	Ref.
Yes	Yes	10	1.97 ± 1.16	− 0.02	0.35	0.951	− 0.26	0.40	0.505
No	No	20	2.31 ± 1.11	0.33	0.27	0.224	0.24	0.31	0.446
No	Yes	55	2.58 ± 1.17	0.59	0.20	0.003	0.68	0.24	0.005
Interaction						0.526			0.184

^a^Adjusted for glycated hemoglobin, triglyceride, low-density lipoprotein

*SD* standard deviation

## Discussion

Testosterone, known as the primary male sex hormone, was suggested that it possesses immunomodulatory properties and affect the occurrence and development of chronic inflammation. In vitro evidence suggests that testosterone inhibits the expression of the inflammatory cytokines, such as TNF-α, IL-1β and IL-6, and enhances the expression of the anti-inflammatory cytokine IL-10 [25]. Animal study disclosed that testosterone deficiency can elicit a state of low-grade inflammation, shown by an increase in IL-6 and TNF-α level, which was consistent with the immunosuppressive effects of testosterone [26–28]. Several epidemiological studies revealed that testosterone deficiency was reported to be linked to a number of inflammatory associated conditions such as MS, T2DM, carotid intimal thickening, aorta, lower extremity arterial disease and others [29–31]. As compared to men with normal TT and FT levels, men with insufficient TT or FT would like to have significantly higher levels of TNF-α and macrophage inflammatory protein 1α (MIP1α) [32]. Meanwhile, testosterone replacement therapy was also reported to be able to largely improve inflammation status. In a randomized placebo-controlled trial to evaluate the effect of testosterone replacement with 250 mg/2 weeks for 24 weeks on insulin resistance in men with T2DM and hypogonadotropic hypogonadism [33], insulin sensitivity was significantly improved in testosterone treatment group, while the concentration of inflammatory markers including CRP, IL-1β, and TNF-α were obviously decreased as compared to the placebo group (*P* < 0.05). In another randomized, single-blind, placebo-controlled clinical trial, testosterone supplement therapy was reported to be able to significantly reduce the levels of TNF-α (*P* = 0.01) as well as IL-1β (*P* = 0.08), and increase IL-10 level (*P* = 0.01) [17]. The potential mechanism might be that testosterone could inhibit chronic inflammation mainly by restricting the expression and release of inflammatory factors and promoting the synthesis and secretion of anti-inflammatory factors [26]. However, there also existed inconsistent findings. Animal studies reported that no differences of the concentration of IL-6 in plasma and tissue were observed between testosterone supplement group and the counterpart [34, 35]. Another study also reported that supplementation of exogenous testosterone did not alter the levels of cytokines in the body [26].

Plasma testosterone mainly existed in three forms: FT, testosterone bound to SHBG, and testosterone bound to albumin. BIOT consists of FT and testosterone bound to albumin and believed as the major bioactive form of testosterone. Consistent with previous studies [17, 28, 32, 33], after adjusting for the confounding factors screened by stepwise regression model, an obvious linear trend of the negative relationship between NLR level and serum BIOT was observed (*P*_trend_ = 0.027). Our findings suggested that elevated intensity of BIOT was significantly associated with decreased level of CSI.

Previous study suggests that the anti-inflammatory ability decreases gradually with aging [36]. As a main target of oxidative damage, mitochondria are believed to play an important role in the process of aging. Accumulating evidences suggest that oxidative stress can induce mitochondrial injury and promote the production of reactive oxygen species, which will further cause lipid peroxidation, nucleic acid oxidation, stress proteins and enzyme inactivation [37–40]. Mitochondrial dysfunction is reported to be significantly associated with age-related diseases such as chronic inflammation, neurological diseases and cancer [30]. It is currently believed that there exists a continuous decrease of testosterone level in men over 45 years old, and testosterone may inhibit the expression and release of cytokines and inflammatory chemotactic factors [17, 25]. Besides, aging is reported to play an important role in the development of chronic inflammation, especially oxidative stress mediated, such as adipose tissue, testicular, thyroid and adrenal gland [41]. Indirectly through the pathways of cell damage, mitochondrial dysfunction and mircoRNA generation, aging-induced oxidative stress and excessive reactive oxygen species is believed to induce increased secretion of inflammatory medium and decreased antioxidant function [42], which eventually lead to endocrine organ inflammation, the reduction of endocrine hormone secretion, hormone anti-aging dysfunction and others [42]. The inflammatory senescence effect may be related to the oxidative stress status and inflammation in the regional anatomy rather than to measurable levels of inflammatory cytokines in the body [21].

In the current study, based on the results of multiple LOESS and GLMs models, an obvious linear trend of positive relationship was observed between the NLR level and age (*P*_trend_ = 0.027), which was consistent with another study [36]. The adjusted elevation of NLR averagely achieved 0.23 with per standard deviation increase of age (*P* = 0.043), which indicated that the inflammation would like to be more obvious as age increased. Our further analysis on the joint association of BIOT and age with NLR disclosed that men elder than 70 years old combined with lower BIOT concentration (BIOT < 4.7 nmol/L) would have the highest level of NLR level as compared to men under 70 years with higher BIOT concentration (BIOT ≥ 4.7 nmol/L), suggesting that aging and lower serum BIOT significantly and jointly correlated with elevated CSI (*P* = 0.005). However, no significant interaction between aging and serum BIOT concentration on NLR was observed in this study (*P* = 0.184).

### Strength and limitations

The main strength of the present study is that the individual and joint associations of BIOT and aging with CSI were comprehensively estimated with adjusting for some potential confounding factors screened by stepwise regression model. (1) Multiple LOESS models, the major component of semiparametric regression approaches, were applied to fully assess the relationships between NLR and BIOT as well as aging rather than routine multiple linear regression models since the relationships between the predictors and response variables were nonlinear in most conditions. Semiparametric regression is a series of novel statistical approaches developed in the past 2 decades, which mainly combines parametrically modeled effects for some predictors with nonparametric modeling of the effects of the other variables [43]. Because of its flexibility, semiparametric regression has been proven to be of great value in many applications in fields as diverse as astronomy, biology, medicine, economics, and finance [43]. This indicated that our results would be reliable and robust. (2) GLMs were performed to extensively and intensively investigate the independent association of NLR with BIOT as well as aging in the following three ways: with exposure as a categorical variable (quartiles for BIOT and three categories for age), as a dichotomous variable (BIOT < 4.7 nmol/L and age ≥ 70 years), and as a continuous variable [scaled to standard deviation (SD) for both BIOT and age]. In addition, the joint association of BIOT and aging on the level of NLR was examined at one time with high BIOT defined as greater than the median exposure (4.7 nmol/L) and aging with under 70 years old as the reference group, rather than only investigating their individual effects one by one. This strategy will be very beneficial in the early identification of a person with CSI or at high risk since it can largely increase the sensitivity and specificity as compared to a single marker. Moreover, to the best of our knowledge, this may be the first study to individually and jointly investigate the relationship between CSI and BIOT or aging. Our findings have implications for the secondary prevention of CSI.

The present study also had limitations. First, the data of this study were only collected from a single hospital and will be unavoidable to induce selection bias to some extent. However, the participants were enrolled from the routine physical examination of middle-aged and older population in the first affiliated hospital of USTC, which is one of the top hospitals of Anhui province and covers a large amount of the residents. The potential selection bias in the current study will not be too obvious as usual. Second, our results were based on a cross-sectional study; it might to be thought as another limitation. In fact, as the basis of many other studies containing case–control study, cohort study and experimental study, a cross-sectional study has been proven to be of great value in many applications in fields as diverse as astronomy, biology, medicine, economics, and finance [43, 44]. A well-designed cross-sectional study is enough to disclose the associations between variables though it will not lead to a clear cause-and-effect relationship among variables. In the present study, we aimed to investigate the individual and joint associations of testosterone and aging with CSI rather than exploring the potential causality between them. So, depending on the high quality of data and a series of reasonable statistical methods, we do think that our findings are reliable since a cross-sectional study is enough to support the association assessment.

## Conclusion

In the present study, we observed that BIOT and aging are individually and jointly associated with NLR in Chinese middle-aged and elderly men. Older males with lower BIOT were more like to having CSI or at high risk than others. To the best of our knowledge, this is the first report to individually and jointly investigate the relationship between CSI and BIOT or aging. Further large longitudinal population-based studies or prospective cohort study will be needed to confirm our conclusion.
